# PLIERv2: bigger, better and faster

**DOI:** 10.1101/2025.06.05.658122

**Published:** 2025-06-08

**Authors:** Marc Subirana-Granés, Sutanu Nandi, Haoyu Zhang, Maria Chikina, Milton Pividori

**Affiliations:** Department of Biomedical Informatics, University of Colorado Anschutz Medical Campus, Aurora, CO, USA; Department of Pharmacology, University of Colorado Anschutz Medical Campus, Aurora, CO, USA; Department of Biomedical Informatics, University of Colorado Anschutz Medical Campus, Aurora, CO, USA; Department of Computational and Systems Biology, University of Pittsburgh School of Medicine, Pittsburgh, PA 15213, USA; Department of Biomedical Informatics, University of Colorado School of Medicine, Aurora, CO 80045, USA; Colorado Center for Personalized Medicine, University of Colorado Anschutz Medical Campus, Aurora, CO, USA

## Abstract

Gene expression analysis has long been fundamental for elucidating molecular pathways and gene–disease relationships, but traditional single-gene approaches cannot capture the coordinated regulatory networks underlying complex phenotypes; although unsupervised matrix factorization methods (e.g., PCA, NMF) reveal coexpression patterns, they lack the ability to incorporate prior biological knowledge and often struggle with interpretability and technical noise correction. Semi-supervised strategies such as PLIER have improved interpretability by integrating pathway annotations during latent variable extraction, yet the original PLIER implementation is prohibitively slow and memory-intensive, making it impractical for modern large-scale resources like ARCHS4 or recount3. Here, we introduce PLIERv2, which overcomes these constraints through a two-phase algorithmic design (an unsupervised “PLIERbase” initialization followed by a “PLIERfull” regression that incorporates priors via glmnet), rigorous internal cross-validation to tune regularization parameters for each latent variable, and efficient on-disk data handling using memory-mapped matrices from the bigstatsr package. Benchmarking on GTEx, recount2, and ARCHS4 demonstrates that PLIERv2 achieves 7×–41× speedups over PLIERv1, succeeds in modeling hundreds of thousands of samples that PLIERv1 cannot handle, and maintains or improves biological specificity of latent variables as shown by tissue-alignment and pathway enrichment analyses. By filling the gap in scalable, biologically informed latent variable extraction, PLIERv2 enables comprehensive analysis of modern transcriptomic compendia and paves the way for deeper insights into gene regulatory networks and downstream applications in translational genomics.

## Introduction

Gene expression analysis has been fundamental for elucidating molecular pathways and understanding gene–disease relationships. Traditionally, insights into biological functions have emerged primarily through single-gene analyses, which identify individual genes whose expression differs between conditions. However, biological processes arise from complex interactions among numerous genes operating in coordinated regulatory networks [1]. Therefore, fully unraveling cellular mechanisms requires methods that can capture not only differential expression but also coexpression patterns across entire transcriptomes.

High-dimensional gene expression data inherently contains correlated structures, reflecting coordinated transcriptional regulation or variations in cell-type composition within heterogeneous tissue samples. Recognizing and exploiting these correlations provides an opportunity to infer regulatory circuits, activated pathways, and cell-type dynamics underlying specific biological states or phenotypes. Furthermore, high-dimensional data often include technical variations or “batch effects” [2], making it crucial to distinguish meaningful biological signals from technical noise.

Recent advances in unsupervised machine learning techniques have been developed, including matrix factorization approaches that capture complex gene expression patterns into more interpretable latent variables (LVs) or gene modules, thereby enhancing biological interpretability [3]. These matrix factorization methods outperform traditional unsupervised clustering by effectively capturing local coexpression patterns present only in subsets of samples and allowing genes to participate in multiple modules simultaneously [3]. While simple linear models such as principal component analysis (PCA) or non-negative matrix factorization NMF provide basic dimensionality reduction, their inability to incorporate prior biological knowledge often limits interpretability and biological relevance [4,5].

To overcome these limitations, semi-supervised decomposition approaches, such as Pathway-Level Information Extractor (PLIER) [6] and GenomicSuperSignature [7], integrate prior biological knowledge into their decomposition frameworks. PLIER combines unsupervised decomposition (via non-negative matrix factorization) with prior pathway annotations directly during the learning process, generating highly interpretable latent representations while effectively separating technical artifacts [6].

PLIER-based methodologies have demonstrated broad applicability and success across diverse biological contexts. For instance, MultiPLIER leveraged large public expression compendia to infer latent variables transferable to smaller datasets, significantly enhancing the identification of gene modules relevant to rare diseases where sample size is limited [8]. MousiPLIER successfully adapted the PLIER framework for use in mouse models, extending its utility beyond human studies and facilitating comparative research across species [9]. By integrating genetic data with gene expression modules, PhenoPLIER combined genome-wide and transcriptome-wide association studies (GWAS and TWAS) with expression-derived LVs, improving mechanistic insights into complex human traits [10]. Finally, OmniPLIER extracted gene modules using RNA-seq data from the Human Trisome Project [11,12] to better understand the molecular interplay between Down syndrome and obesity [13].

Despite its widespread applicability and success, the original implementation of PLIER presents notable limitations. One critical issue is computational performance: PLIER can be prohibitively slow, limiting scalability to large datasets. Consequently, analyzing large-scale resources such as ARCHS4 [14] or recount3 [15], which contain tens of thousands of samples, is currently impractical due to excessive memory demands and computational runtimes.

To overcome these constraints, we introduce PLIERv2, a significantly optimized implementation designed specifically to handle modern large-scale transcriptomic datasets efficiently and with extensive biological priors without sacrificing computational precision.

## Software description

PLIERv2 builds on PLIER1 with significant enhancements in both algorithm design and data handling, yielding faster performance and improved scalability.

### Algorithmic improvements

Given an input gene by sample matrix Y and a gene by gene-set prior information matrix C the PLIER framework solves the following optimization problem

$$\begin{array}{c} \underset{Z,B,U}{min}\;\|Y-ZB{\|}_{F}^{2}+\lambda_{1}\|Z-CU{\|}_{F}^{2}+\lambda_{2}\|B{\|}_{F}^{2}+\lambda_{3}\|U{\|}_{L^{1}/L^{2}}\\ \text{subject to}\;U>0,\;Z>0 \end{array}$$

Where we use the Z>0 as shorthand for $z_{i,j}\geq 0$.

The objective function includes a reconstruction loss on Y, a prior-induced regularization on Z, ridge regularization on B, and a sparsity-inducing group lasso penalty on U; the hyperparameters $\lambda_{1}$ and $\lambda_{2}$ are automatically determined based on the spectral properties of the data.

A key innovation in PLIERv2 is the explicit separation of two computational phases: PLIERbase and PLIERfull. This phase captures the rapid early changes in latent variables. Since prior information has little effect on gradients during this stage, we omit it, extending the insight from PLIER1, which hardcoded this exclusion for the first 30 iterations. In PLIERv2, this is made explicit and configurable, and the base phase is run to convergence rather than stopping after an arbitrary number of steps.

PLIERbase runs the PLIER factorization without prior information, but retains the same $\lambda_{2},\;\lambda_{1}$ regularization and non-negativity constraints on the loadings matrix (Z). The formal PLIERbase problem is:

$$\begin{array}{c} \underset{Z,B}{min}\;\|Y-ZB{\|}_{F}^{2}+\lambda_{1}\|Z{\|}_{F}^{2}+\lambda_{2}\|B{\|}_{F}^{2}\\ \text{subject to}\;Z>0 \end{array}$$

In the second phase, PLIERfull, prior knowledge is introduced via a regression framework that models Z as a function of the prior information matrix U. This is implemented using glmnet, which efficiently solves the underlying L1/L2 regularized regression.

A key improvement in PLIERv2 is how the regularization strength, $\lambda_{3}$, is selected. Whereas PLIER1 adjusted this parameter iteratively to meet a fixed target (e.g., 70% of latent variables associated with pathways), PLIERv2 adopts a more rigorous approach using internal cross-validation. Specifically, each latent variable is assigned an individualized $\lambda_{3}$ via cv.glmnet. For efficiency, the search is restricted to 20 candidate values by default, and the optimization is performed independently for each latent variable. This allows the model to automatically determine whether or not to associate prior information with each latent variable—some or all LVs may be linked to pathways, or none at all. To reduce attenuation bias, the selected pathway coefficients are refit using unregularized regression. Finally, since the U coefficients tend to change slowly, we update them only every other iteration and cap the number of updates using a max.U.updates parameter (default: five iterations).

**Figure 1:**
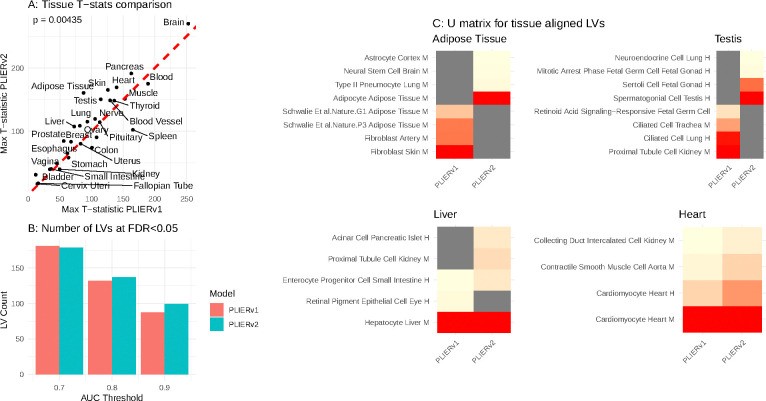
Comparative Tissue Alignment and Prior-Knowledge Integration in PLIERv1 versus PLIERv2. **(A)** Scatter plot comparing the maximum tissue-specific T-statistic achieved by any latent variable (LV) for each of 54 GTEx tissues between PLIERv1 (x-axis) and PLIERv2 (y-axis). Each point corresponds to a single tissue, with larger T-statistics indicating stronger alignment of an LV to that tissue annotation. PLIERv2 showed a significant overall improvement in tissue alignment p-value (p = 0.00435, paired rank-sum test). **(B)** Bar plot showing the count of LVs with Benjamini–Hochberg FDR < 0.05 at cross-validated AUC thresholds of 0.7, 0.8, and 0.9 using GTExV8. Bars colored in salmon denote PLIERv1, while bars in teal denote PLIERv2. At higher AUC cut-off (0.8, 0.9), PLIERv2 yields more high-confidence LVs than PLIERv1, indicating stronger and more numerous links to prior biological knowledge. **(C)** Heatmaps of U-matrix coefficients for representative tissue-aligned LVs with improved PLIERv2 performance. Within each tissue panel, the left column shows the normalized coefficient values of the top four prior gene-sets associated with the highest-scoring LV from PLIERv1, while the right column shows the corresponding values for PLIERv2. Row labels indicate the four highest-weighted prior gene-sets (e.g., “Adipocyte Adipose Tissue M” vs. “Fibroblast Skin M” in Adipose Tissue; “Spermatogonial Cell Testis H” vs. “Proximal Tubule Cell Kidney M” in Testis). In some cases the pathways selected are highly similar (Liver and Heart), though the coefficients differ. In other cases (Testis and Adipose) PLIERv2 clearly identified more biologically specific associations (for example, adipocyte markers in adipose tissue and spermatogonial markers in testis) compared to PLIERv1.

The final latent variable–pathway associations are validated using an outer cross-validation step. Specifically, we withhold all annotations for 10% of the genes during model fitting and assess whether these can be recovered through the inferred LV loadings (columns of the Z matrix). This procedure yields well-calibrated AUCs, p-values, and FDRs based on rank-sum testing. This outer cross-validation approach, originally introduced in PLIER1, provides a principled measure of biological relevance. We also use it to evaluate the improvements in PLIERv2 and find that, as expected, incorporating internal cross-validation enhances performance in the outer validation (Figure 1c).

Finally, we have enhanced the algorithm’s data handling capabilities to support large, on-disk datasets. This is achieved using memory-mapped file infrastructure through **FBM** (Filebacked Big Matrix) objects provided by the **bigstatsr** package. Unlike general-purpose on-disk format, **FBM** objects are specifically designed to support efficient linear algebra operations by enabling memory-mapped access and integrating tightly with optimized statistical routines in **bigstatsr**. These files are also highly interoperable—compact and transparent enough to be written and accessed directly from other computing environments such as Python.

## Results

### PLIERv2 recapitulates PLIER-derived latent variables across GTEx while improving disentanglement performance

To evaluate whether the updated PLIERv2 algorithm both replicates and improves upon PLIERv1, we retrained both models on the GTEx v8 compendium (17,382 RNA-seq profiles across 54 tissue types) using identical inputs: the same pre-computed SVD, an identical gene set prior, and the same number of latent variables (k = 500). Our primary focus was identifying latent variables (LVs) that are strongly associated with tissue annotations. As prior knowledge, we used the CellMarker2024 gene set downloaded from Enrichr [16]. We quantify the tissue alignment for an LV by reporting the T-statistic for the comparison of samples belonging to that tissue with the rest. For each tissue, we record the maximal T-statistic value, allowing for comparisons across models with the same number of LVs. All the T-statistics are highly significant and we omit the p-values.

We find that PLIERv2 consistently achieves significantly better alignment (Figure 1a). Inspection of the pathway associations for LVs with improved alignment reveals that while some tissues (e.g., heart, liver) show similar pathway support across models, the more pronounced differences in other tissues correspond to biologically more meaningful associations in PLIERv2. Examples include Adipocyte tissue being associated with “Adipocyte Adipose Tissue Mouse“ rather than Fibroblasts and Testis tissue being associated with”Spermatogonial cell, testis” (Figure 1c).

Finally, using outer cross-validation, we check if genes whose annotations were dropped can be classified by their loading values. For each LV, we record the maximal cross-validation AUC it achieves for any of the associated pathways. Retaining only the ROC curve (AUC) for gene-set enrichment that passed Benjamini–Hochberg FDR < 0.05, PLIERv2 produced more latent variables at higher AUC thresholds (0.8 and 0.9) than PLIERv1, underscoring its stronger and more numerous links to prior biology (Figure 1b). Altogether, these results demonstrate that PLIERv2 more effectively aligns co-expressed LVs with prior biological knowledge.

### PLIERv2 exhibits superior computational efficiency and enables modeling of large-scale ARCHS4 data

**Figure 2:**
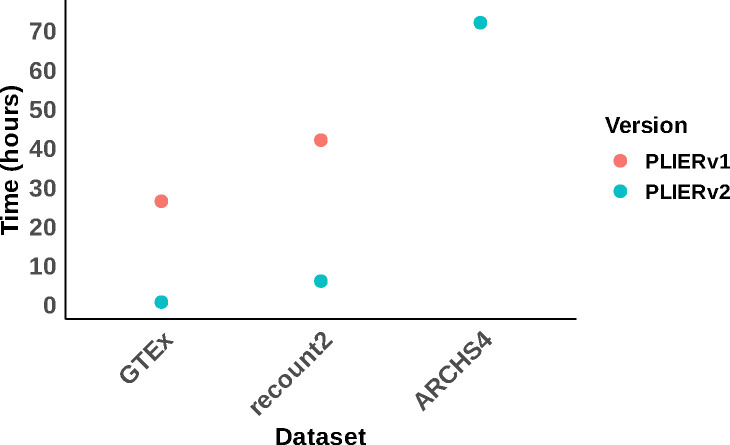
Comparative Computational time benchmarking of PLIERv1 and PLIERv2 across datasets of different sizes. Wall-clock time (in hours) for PLIERv1 (salmon) and PLIERv2 (teal) is shown on three transcriptomic compendia ordered by increasing size (from left to right: GTEx v8, recount2, ARCHS4). Each dot represents an independent run on that dataset, illustrating that PLIERv2 consistently reduces computational time as dataset size grows. PLIERv2 is the only algorithm capable of generating a full ARCHS4 model using all samples.

We systematically benchmarked the computational performance of PLIERv1 and the optimized PLIERv2 across three large-scale human transcriptomic compendia (GTEx, recount2, and ARCHS4) by quantifying total model runtime in hours under standardized hardware conditions. All benchmarking analyses were conducted on a dedicated workstation equipped with an Intel^®^ Xeon^®^ w5-2465X processor (32 physical cores) and 256 GB RAM, providing sufficient computational resources for high-dimensional matrix decomposition tasks. As shown in (Figure 2), PLIERv2 consistently and substantially outperforms PLIERv1 across all datasets. On the GTEx v8 compendium (~17K RNA-seq samples with ~56K Genes), PLIERv1 required approximately 26.4 hours, while PLIERv2 completed the task in just 0.64 hours, corresponding to a ~41× speedup. On the recount2 dataset (~30K samples of uniformly processed human RNA-seq with ~30K genes), PLIERv1 executed in ~42.0 hours, compared to only ~6.0 hours for PLIERv2, yielding a ~7× improvement. For the ARCHS4 dataset (~600K RNA-seq samples with ~20K genes), PLIERv1 failed to complete due to computational limitations, whereas PLIERv2 successfully executed the analysis in ~72.0 hours. These benchmarking results clearly demonstrate that PLIERv2 offers dramatic improvements in computational efficiency, with speedups ranging from ~7× to ~41× depending on dataset size. Additionally, the progressive increase in runtime from GTEx to ARCHS4 observed in PLIERv2 aligns with expected increases in data dimensionality and complexity, and underscores its robust scalability for large-scale transcriptomic analyses.

## Discussion

A central challenge in large-scale transcriptomic analyses is accurately inferring biologically meaningful signatures, such as variation in cell-type proportions or pathway activity, from global gene expression profiles while simultaneously mitigating technical noise and avoiding prohibitive computational costs. The original PLIER algorithm addressed part of this problem by integrating prior biological knowledge into LV extraction, thereby enhancing interpretability. However, its practical utility was constrained by scalability limitations, which became especially noticeable when attempting to analyze very large compendia such as ARCHS4 or recount3.

In this study, we introduced PLIERv2, which directly addresses these limitations through strategic algorithmic innovations and optimized computational handling. Our comprehensive benchmarking demonstrates that PLIERv2 achieves significantly improved computational efficiency, effectively scaling to large transcriptomic datasets previously inaccessible to the original algorithm. Specifically, by explicitly separating the computational phases into PLIERbase (an unsupervised initialization) and PLIERfull (integration of prior knowledge), we minimized computational redundancies and improved convergence behavior. The use of nested cross-validation to rigorously tune regularization parameters further enhanced both computational efficiency and biological interpretability.

Beyond computational improvements, our evaluations across GTEx datasets demonstrated that PLIERv2 not only replicates latent variables identified by its predecessor but also markedly improves biological specificity. PLIERv2 yielded high-confidence associations between latent variables and known biological pathways or cell types, improving the interpretability of unsupervised transcriptomic analyses. It also showed tissue-specific LV alignment scores significantly increased, reflecting better capture of biologically relevant gene sets. In particular, tissues such as adipose and testis showed enhanced biological coherence in their associated LVs when analyzed with PLIERv2.

Moreover, PLIERv2 significantly reduced computational time compared to the original implementation, dramatically improving performance. Crucially, PLIERv2 successfully overcame the prior limitations of generating models on extensive datasets like ARCHS4. This improvement allows researchers to efficiently analyze large-scale transcriptomic data, expanding the potential applications.

In conclusion, PLIERv2 improvements in computational efficiency and biologically informed latent variable extraction represent a substantial step forward in bioinformatics tools for large transcriptomic data analysis, facilitating deeper insights into gene regulatory networks and biological processes. Future work could focus on integrating PLIERv2 within broader multi-omics frameworks, potentially expanding its utility across diverse genomic and clinical research applications.

## Data Availability

PLIERv2 is implemented as an R package supported on Linux. PLIERv2 is available from GitHub (https://github.com/pivlab/plier2).
